# Development of a Liquid Chromatography–Tandem Mass Spectrometry Method for Oxylipin Analysis and Its Application to Children’s Plasma

**DOI:** 10.3390/diagnostics15151870

**Published:** 2025-07-25

**Authors:** Yonghan Li, Siddabasave Gowda B. Gowda, Divyavani Gowda, Atsuko Ikeda, Yu Ait Bamai, Rahel Mesfin Ketema, Reiko Kishi, Hitoshi Chiba, Shu-Ping Hui

**Affiliations:** 1 Graduate School of Health Sciences, Hokkaido University, Kita-12, Nishi-5, Kita-ku, Sapporo 060-0812, Japan; liyonghan846@gamil.com; 2 Graduate School of Global Food Resources, Hokkaido University, Kita-9, Nishi-9, Kita-Ku, Sapporo 060-0809, Japan; sbgowda100@gmail.com; 3 Faculty of Health Sciences, Hokkaido University, Kita-12, Nishi-5, Kita-ku, Sapporo 060-0812, Japan; divyavanisbgowda@gmail.com (D.G.); atsuko_ikeda@hs.hokudai.ac.jp (A.I.); krahel@cehs.hokudai.ac.jp (R.M.K.); 4 Center for Environmental and Health Sciences, Hokkaido University, Kita-12, Nishi-7, Kita-ku, Sapporo 060-0812, Japan; u-aitbamai@med.hokudai.ac.jp (Y.A.B.); rkishi@med.hokudai.ac.jp (R.K.); 5 Department of Nutrition, Sapporo University of Health Sciences, Nakanuma Nishi-4-2-1-15, Higashi-ku, Sapporo 070-0894, Japan; chibahitgm@gmail.com

**Keywords:** oxylipins, polyunsaturated fatty acids, plasma, children, liquid chromatography, mass spectrometry

## Abstract

**Background/Objectives:** Oxylipins, a family of oxygenated natural products derived from polyunsaturated fatty acids (PUFAs), play crucial roles in various physiological processes. Evaluating their levels in vivo helps to reveal their roles in health and disease. Because of the numerous isomers of oxylipins, it is essential to develop efficient and precise analytical methods for their identification and quantification. The objective of this study is to establish a quantitative method for oxylipin analysis and its application to the assessment of oxylipins in children’s plasma, with potential implications for diagnostic use in pediatric populations. **Methods:** A liquid chromatography–electrospray ionization–tandem mass spectrometry method was developed to quantify 64 oxylipins and four precursor PUFAs within 36 min. The limits of quantification ranged from 0.25 to 50 pg, with most analytes showing recoveries and matrix effects between 85 and 110% and between 90 and 110%, respectively. Intra- and inter-day precision values were within 15%. The established method was applied to plasma samples from children aged 9–12 years (boys = 181; girls = 161) in Hokkaido, Japan, to assess the relation between plasma oxylipin and PUFA levels and age, sex, and body mass index. **Results:** There was no significant correlation between oxylipin levels and age, sex, or body mass index. However, among the PUFAs, boys had higher eicosapentaenoic acid and arachidonic acid levels than those of girls, with a significant increase in eicosapentaenoic acid levels in the overweight group compared with those in the underweight group. **Conclusions:** We successfully developed a simple and highly selective method for the analysis of oxylipins in preadolescent children’s plasma samples. Thus, this study provides a foundation for broader application of the developed method to different biological samples in future studies.

## 1. Introduction

**Figure 1:**
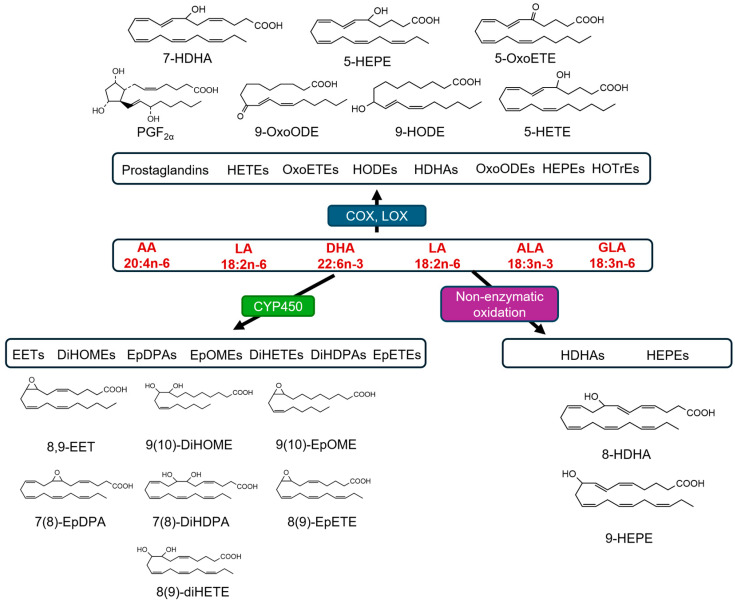
Conversion of PUFAs (AA, LA, DHA, and EPA) into oxylipins. DiHOME: Dihydroxy-octadecenoic acid; HODE: hydroxy-octadecadienoic acid; EPA: eicosapentaenoic acid; LA: linoleic acid; AA: arachidonic acid; PUFA: polyunsaturated fatty acid; CYP450: cytochrome P450; OxoETE: oxo-eicosatetraenoic acid; HETE: hydroxy-eicosatetraenoic acid; DiHDPA: dihydroxy-docosapentaenoic acid. “20:4 n-6” refers to a 20-carbon omega-6 fatty acid with four double bonds and “22:6 n-3” refers to a 22-carbon omega-3 fatty acid with six double bonds.

Oxylipins are a diverse and biologically significant family of oxygenated fatty acids derived from polyunsaturated fatty acids (PUFAs), such as arachidonic acid (AA), eicosapentaenoic acid (EPA), docosahexaenoic acid (DHA), and linoleic acid (LA) [1,2,3]. The metabolic transformation of PUFAs into oxylipins occurs via several enzymatic pathways, including cyclooxygenases, lipoxygenases, and cytochrome P450 monooxygenases, as illustrated in Figure 1. Bioactive lipids play crucial roles in various physiological and pathophysiological processes, making them essential for biomedical research [1]. The biological importance of oxylipins is underscored by their involvement in regulating inflammation, immunity, and vascular function [1]. Oxylipins can act as signaling molecules that modulate inflammatory responses. For instance, prostaglandins and leukotrienes, which are derived from AA through enzymatic pathways involving cyclooxygenase and lipoxygenase, respectively, are well-known mediators of inflammation and allergic reactions that contribute to the onset and resolution of inflammation [4]. Oxylipins play a significant role in maintaining vascular homeostasis. Epoxyeicosatrienoic acids (EETs), produced by the cytochrome P450 pathway, exhibit vasodilatory properties and contribute to blood pressure regulation. They also possess anti-inflammatory and cardioprotective properties, which highlight their therapeutic potential against cardiovascular diseases [5]. Furthermore, the dysregulation of oxylipin pathways has been implicated in various diseases, including asthma, cardiovascular diseases, cancer, and neurodegenerative disorders. Elevated levels of proinflammatory oxylipins, such as leukotrienes, have been associated with the pathogenesis of asthma and other allergic conditions [6]. Similarly, alterations in the balance between pro- and anti-inflammatory oxylipins have been observed in cardiovascular diseases, contributing to endothelial dysfunction and atherosclerosis [7].

The detection and quantification of oxylipins in plasma are particularly important for pediatric populations, as it aids in the early diagnosis and monitoring of pediatric diseases characterized by inflammation and immune dysregulation, such as asthma, allergies, and autoimmune diseases [8,9]. Additionally, monitoring oxylipins in pediatric plasma enhances our understanding of the potential of dietary interventions and pharmacological treatments [10,11]. Studies have indicated that n-3 (also known as ω-3) fatty acids such as ALA, EPA, and DHA (those having cis-double at C-3 carbon from the end terminal of fatty acids), which are precursors of anti-inflammatory oxylipins, modulate the oxylipin profile and offer protective effects against inflammation and immune dysregulation in children [10,11]. By quantifying oxylipin levels before and after dietary or drug interventions, researchers can evaluate the efficacy of these strategies and optimize treatment plans for pediatric populations.

It is evident in the literature that gas chromatography–mass spectrometry (GC-MS) technique has been used for the analysis of oxylipins [12,13]. However, GC-MS requires extensive sample preparation, including derivatization (typically involving the conversion of polar functional groups into more volatile and thermally stable derivatives that are compatible with gas-phase separation), which can be time-consuming and labor-intensive [14]. Recently, liquid chromatography–mass spectrometry (LC-MS) has emerged as a powerful alternative with several advantages over GC-MS. LC-MS eliminates the need for derivatization, thereby simplifying sample preparation and reducing the risk of oxylipin degradation [15,16]. Furthermore, coupling liquid chromatography with tandem mass spectrometry has significantly enhanced the specificity and sensitivity of oxylipin detection [17,18,19]. This technique allows the simultaneous quantification of multiple oxylipins in complex biological matrices, providing a comprehensive profile of these bioactive lipids [17,18,19].

Oxylipins are key mediators of inflammation, immunity, and vascular function. Establishing reliable and sensitive analytical methods is essential for elucidating their biological roles in the human body. Despite their recognized significance, current studies lack simple extraction and comprehensive oxylipin profiling in pediatric populations. In this study, we developed and optimized an extremely sensitive and selective method for oxylipin detection using liquid chromatography–tandem mass spectrometry coupled with multiple reaction monitoring (MRM). Understanding the levels and composition of oxylipins in children during this critical developmental period is crucial for elucidating the potential impact of these lipid mediators on child health and development. Our study not only fills a gap in the current research but also provides foundational data and novel methodologies for future investigations into the roles of oxylipins in pediatric diseases and health conditions.

## 2. Materials and Methods

### 2.1. Materials

The following authentic standards were obtained from Cayman Chemical (Ann Arbor, MI, USA): (±)12(13)-dihydroxy-octadecenoic acid ((±)12(13)-DiHOME), (±)9(10)-DiHOME, 9(S)-hydroxy-octadecadienoic acid (9(S)-HODE), 13(S)-HODE, 13-oxo-octadecadienoic acid, 9-oxo-octadecadienoic acid, (±)12(13)-epoxy-octadecenoic acid, (±)9(10)-epoxy-octadecenoic acid, (±)19(20)-dihydroxy-docosapentaenoic acid ((±)19(20)-DiHDPA), (±)16(17)-DiHDPA, (±)13(14)-DiHDPA, (±)10(11)-DiHDPA, (±)7(8)-DiHDPA, (±)19(20)-epoxy-docosapentaenoic acid ((±)19(20)-EpDPA), (±)16(17)-EpDPA, (±)13(14)-EpDPA, (±)10(11)-EpDPA, (±)7(8)-EpDPA, prostaglandin E3, prostaglandin F3α, 5(S)-hydroxy-eicosapentaenoic acid (5(S)-HEPE), (±)8-HEPE, (±)9-HEPE, (±)11-HEPE, 12(S)-HEPE, 15(S)-HEPE, (±)18-HEPE, (±)8(9)-epoxy-eicosatetraenoic acid ((±)8(9)-EpETE), (±)11(12)-EpETE, (±)14(15)-EpETE, (±)17(18)-EpETE, (±)5(6)-dihydroxy-eicosatetraenoic acid ((±)5(6)-DiHETE), (±)11(12)-DiHETE, (±)8(9)-DiHETE, (±)14(15)-DiHETE, (±)17(18)-DiHETE, (±)4-hydroxy-DHA ((±)4-HDHA), (±)7-HDHA, (±)8-HDHA, (±)10-HDHA, (±)11-HDHA, (±)13-HDHA, 14(S)-HDHA, (±)16-HDHA, 17(S)-HDHA, (±)20-HDHA, 9(S)-hydroxy-octadecatrienoic acid (9(S)-HOTrE), 13(S)-HOTrE, 13(S)-HOTrE(γ), (±)5-hydroxy-eicosatetraenoic acid ((±)5-HETE), (±)8-HETE, (±)11-HETE, (±)12-HETE, (±)15-HETE, (±)5(6)-epoxy-eicosatetraenoic acid ((±)5(6)-EET), (±)8(9)-EET, (±)11(12)-EET, (±)14(15)-EET, 5-oxo-eicosatetraenoic acid (5-OxoETE), 12-OxoETE, 15-OxoETE, prostaglandin F2α, (±)5-iso prostaglandin F2α-VI, 8-iso prostaglandin F2α, AA, DHA, EPA, and LA. Deuterium-labelled internal standards, such as 8-iso prostaglandin F2α-d4, AA-d8, 15(S)HETE-d8, 12(S)HETE-d8, and 5(S)HETE-d8, were also obtained from Cayman Chemical (Ann Arbor, MI, USA).

LC-MS-grade methanol and acetonitrile were purchased from Kanto Chemical Co. (Tokyo, Japan). Acetic acid (LC-MS grade) was obtained from the Fujifilm Wako Pure Chemical Corporation (Osaka, Japan). Butylated hydroxytoluene (BHT), which was used as an antioxidant during lipid extraction, was sourced from Tokyo Chemical Industry Co., Ltd. (Tokyo, Japan). All standards were dissolved in methanol and stored at −80 °C to maintain their stability.

### 2.2. Human Samples

Plasma samples were obtained from non-fasting children aged 9–12 years (*n* = 342; boys = 181; girls = 161) who were part of the Hokkaido Study on Environment and Children’s Health, Hokkaido Cohort [20,21,22,23]. The criteria for selecting participants are detailed in separate publications [24,25]. The surveyed children primarily resided in Sapporo, Hokkaido, and its surrounding areas. They were born between April 2006 and January 2010. On the day of the survey, they attended designated clinics with their guardians, where they underwent blood sampling and measurements of height, weight, and other physical parameters. The study’s objectives and methods were thoroughly explained to both the children and their parents. Written informed consent was obtained from all parents, and assent was obtained from all participating children.

### 2.3. Extraction of Oxylipins from Plasma Samples

**Figure 2:**
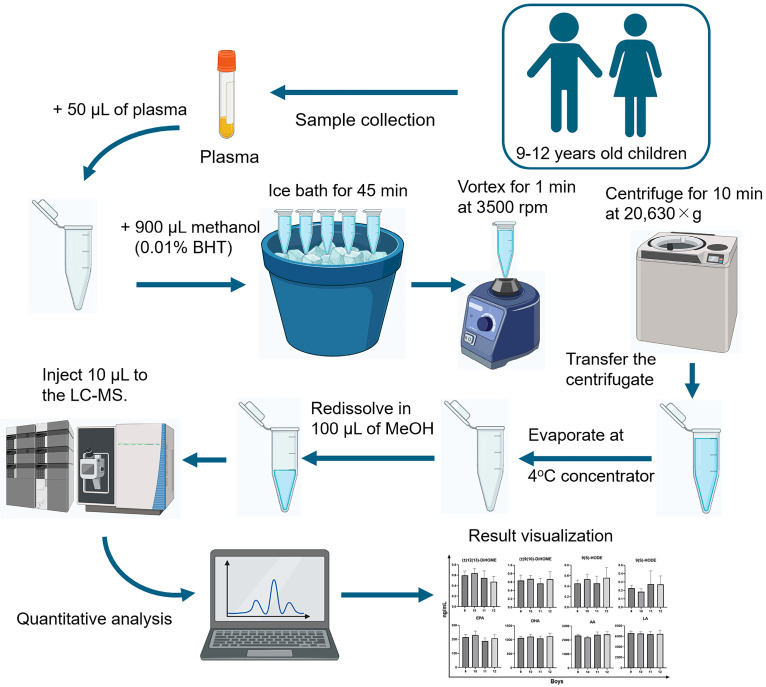
Liquid chromatography–tandem mass spectrometry (LC-MS/MS) methodology for quantitative analysis of children’s plasma samples.

As depicted in Figure 2, a standardized protocol [26] was employed for the quantitative analysis of oxylipin family members in the plasma of children aged 9–12 years. Initially, 50 µL of plasma was mixed with 900 µL of methanol containing 0.01% BHT. This mixture was then incubated in an ice bath for 45 min to precipitate proteins and reduce the enzymatic activity that could degrade oxylipins. Subsequently, the samples were vortexed for 1 min at 3500 rpm to ensure thorough mixing. The samples were then centrifuged at 20,630× *g* for 10 min at 4 °C to separate the supernatant containing the oxylipins. The resulting supernatant was transferred to a new tube and evaporated at 4 °C to concentrate the oxylipins. The dried residue was re-dissolved in 100 µL of methanol to prepare it for analysis. Finally, a 10 µL aliquot of the reconstituted sample was injected into the LC-MS for quantification.

### 2.4. LC-MS Analysis

The LC-MS conditions for analysis were meticulously optimized to ensure high sensitivity and specificity. The liquid chromatography system utilized was the Shimadzu UFLC system (Shimadzu, Kyoto, Japan), equipped with a Kinetex column (Phenomenex Torrance, CA, USA, 100 × 2.1 mm, 2.6 µm). The mobile phase flow rate was set to 0.24 mL/min, with the column temperature maintained at 40 °C. Solvent A consisted of water and 0.1% acetic acid, whereas solvent B consisted of a mixture of methanol and acetonitrile (50/50, *v*/*v*) with 0.1% acetic acid. The injection volume for each sample was 10 µL. The gradient elution program was as follows: starting with 100% solvent A, the composition was maintained for 6 min, and then changed to 43% solvent A and 57% solvent B over the next 3 min, reaching 34% solvent A and 66% solvent B after 20 min, followed by 24% solvent A and 76% solvent B after 22 min. After 27 min, the eluent was 100% solvent B, which was held for 33 min before returning to the initial conditions at 35 min.

For mass spectrometry measurements, a Thermo Scientific TSQ Quantum Access MAX triple-quadrupole system (Thermo Fisher, San Jose, CA, USA) was employed, operating in the negative ionization mode using HESI-II. Acquisition was performed in MRM mode. Key MS parameters included a spray voltage of 4 kV, vaporizer temperature of 250 °C, sheath gas pressure of 30 arbitrary units, auxiliary gas pressure of 5 arbitrary units, and capillary temperature of 300 °C. The collision pressure was set to 1.5 mTorr with a skimmer offset of −5 V. Tube lens and collision energy were compound-specific, ranging from 60 to 92 V and 15 to 27 eV, respectively. The optimized MS parameters for each analyte are listed in Table 1. Nitrogen was used as the desolvation gas, and argon was used as the collision gas. Data acquisition was performed using the Xcalibur 2.2 software.

### 2.5. Linearity and Range

The linearity of the oxylipins was assessed using calibration curves prepared from 12 different concentrations of mixed solutions ranging from 0.001 to 100 ng/mL. For the four precursor PUFAs (AA, DHA, EPA, and LA), calibration curves were generated using 19 different concentrations ranging from 0.001 to 20,000 ng/mL. Each calibration level was prepared with 50 ng/mL of an IS for oxylipins and PUFAs, either 8-iso prostaglandin F2α-d4, AA-d8, 15(S)-HETE-d8, 12(S)-HETE-d8, or 5(S)-HETE-d8, selected based on structural or positional similarity to the corresponding analyte. However, not all concentrations were used for each compound. Calibration curves were constructed by plotting the ratio of the analyte area to the internal standard area against the corresponding concentration ratio. The limit of detection (LOD) was determined based on a signal-to-noise ratio of 3, whereas the limit of quantification (LOQ) was defined using a signal-to-noise ratio of 10.

### 2.6. Accuracy and Precision

To assess the accuracy and precision of the oxylipin quantification, pooled plasma samples were spiked with three concentrations of mixed oxylipin solutions at low (50 ng/mL), medium (75 ng/mL), and high (100 ng/mL) levels (*n* = 4). The intra-day accuracy was determined by calculating the percentage ratio of the measured mean concentration to the expected concentration for each level. Inter-day accuracy was evaluated by calculating the percentage ratio of the mean concentration measured on the second day to the expected concentration for each level. Both intra-day and inter-day precision were expressed as the relative standard deviation (RSD).

### 2.7. Recovery and Matrix Effects

Mixed oxylipin solutions at three different concentrations (low, 50 ng/mL; medium, 75 ng/mL; high, 100 ng/mL) and an IS solution (50 ng/mL) were added to methanol (Group A), pre-extraction pooled plasma (Group B), and post-extraction pooled plasma (Group C), with each group tested in quadruplicate (*n* = 4). Recovery was calculated as the ratio of the mean analyte/internal standard area in Group B to Group A and was expressed as a percentage (B/A × 100%). Matrix effects were assessed by calculating the ratio of the mean analyte/internal standard area in Group C to Group A and were expressed as a percentage (C/A × 100%).

### 2.8. Blood Test Analysis

The relationship between oxylipin and PUFA levels to hematological parameters was assessed through complete blood cell counts, which were performed by Daiichi Kishimoto Clinical Laboratories Co., Ltd. (Sapporo, Japan) in accordance with standardized protocols for processing and reporting.

### 2.9. Data Visualization and Statistics

Statistical analyses and data visualization were performed using GraphPad Prism version 9.5.0 software (San Diego, CA, USA). The Shapiro–Wilk test assessed the normality of oxylipin and PUFA concentrations, demonstrating a non-normal distribution (*p* < 0.05). Subsequent analysis using the Kruskal–Wallis test indicated statistically significant differences among the groups (*p* < 0.05). Dunn’s multiple comparison test was used to further analyze these differences, with *p* < 0.05 denoting statistical significance. The Kolmogorov–Smirnov test was employed to analyze a single variable, with significance set at *p* < 0.05.

## 3. Results and Discussion

### 3.1. Optimization of Mass Spectrometry Parameters and Selection of Product Ions

To the best of our knowledge, there is limited research on analyzing oxylipins in the plasma of healthy prepubescent children. While developing our analytical method for oxylipins, we encountered significant challenges similar to those reported in previous studies [27,28]. Firstly, oxylipins consist of many structurally similar isomers, necessitating precise separation by liquid chromatography and accurate detection by mass spectrometry. This structural similarity requires high-resolution techniques for proper identification and quantification. Secondly, the extremely low endogenous concentrations of oxylipins in biological samples present significant hurdles to method sensitivity, requiring advanced analytical techniques capable of detecting trace levels with high accuracy. Finally, the multiple unsaturated double bonds in oxylipin structures render them highly susceptible to oxidation. Thus, stringent precautions must be taken during sample preparation and analysis to preserve stability and integrity and ensure reliable and reproducible results. In this study, BHT was employed as an antioxidant to mitigate sample oxidation, and extended elution times were implemented to ensure adequate separation of isomers. In addition, more distinctive fragment ions were utilized in the triple-quadrupole mass spectrometer, which enhanced the reliability and reproducibility of the analytical results.

**Figure 3:**
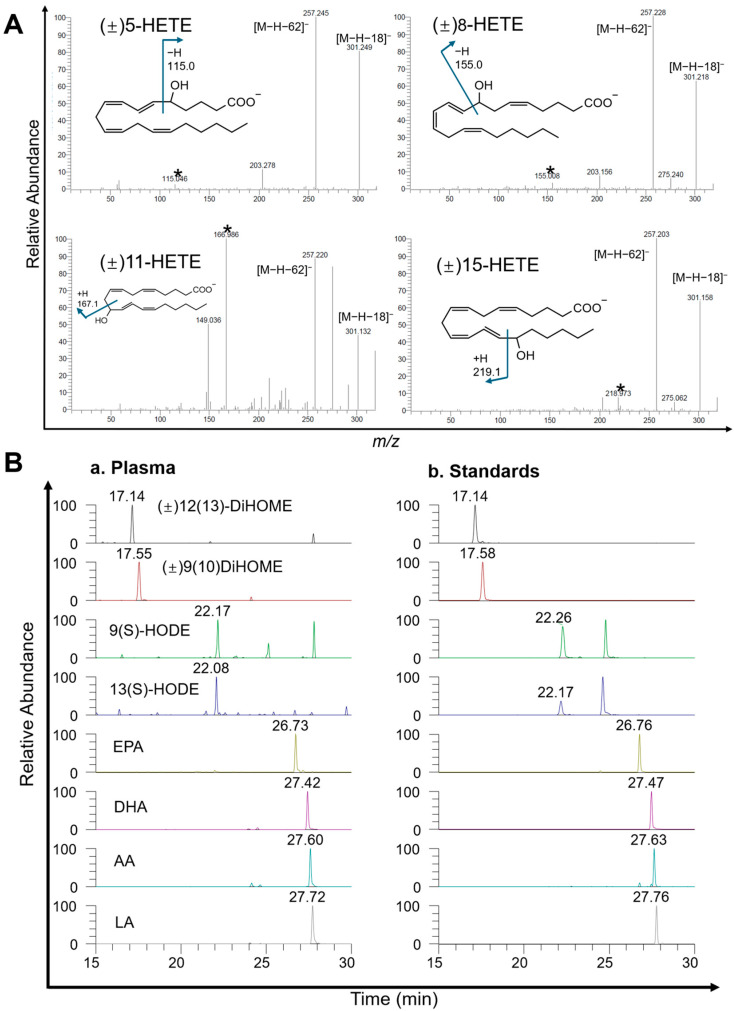
MS/MS spectra and EICs of HETE isomers and oxylipins/PUFAs in children’s plasma samples and standards. (**A**) The MS/MS spectra of four HETE isomers, including (±)5-HETE, (±)8-HETE, (±)11-HETE, and (±)15-HETE, are presented, with each spectrum highlighting the fragmentation pattern and characteristic ions (*), which assist in identifying these isomers based on their distinct fragmentation pathways. (**B**) The EICs of selected oxylipins and PUFAs from plasma samples of children aged 9–12 years (a) and corresponding standards (b) are shown. The compounds analyzed include (±)12(13)-DiHOME, (±)9(10)DiHOME, 9(S)-HODE, 13(S)-HODE, EPA, DHA, AA, and LA. The chromatograms demonstrated the retention times and peak shapes of these analytes, illustrating the ability of this method to resolve and quantify these compounds in biological samples. HODE: Hydroxy-octadecadienoic acid; EPA: eicosapentaenoic acid; LA: linoleic acid; AA: arachidonic acid; PUFA: polyunsaturated fatty acid; HETE: hydroxy-eicosatetraenoic acid; DiHOME: dihydroxy-octadecenoic acid.

In this study, we analyzed 64 oxylipins, including numerous structural isomers with closely related chemical properties, fragmentation patterns, and retention times. For example, within the HETE family, (±)5-HETE, (±)8-HETE, (±)11-HETE, (±)12-HETE, and (±)15-HETE produce a predominant fragment ion [M-18-H]^−^, as shown in Figure 3A, due to the loss of H_2_O from the carboxyl group. Despite this ion’s intensity, their close retention times (23.2–24.5 min) make separation challenging. To enhance detection accuracy, we prioritized more distinctive fragmentation ions over the most sensitive ones to avoid isomer interference, such as 319.2/115.0 for (±)5-HETE and 319.2/219.0 for (±)15-HETE.

### 3.2. Validation of LC-MS Method for Oxylipin Analysis

To ensure the robustness of the analytical method for oxylipin quantification, several validation parameters were evaluated, including retention time, LOD, LOQ, linearity, linear range, and slope, as summarized in Table 2. The retention times were consistent for each oxylipin, indicating reliable chromatographic separation, ranging from 12.36 min for Prostaglandin F3α to 27.81 min for LA. The method demonstrated exceptionally low LODs and LOQs, ranging from 0.1 to 25 pg and 0.25 to 50 pg, respectively, facilitating the detection of trace oxylipin levels. It also exhibited a broad linear range for oxylipin quantification, typically spanning 5–1000 pg, ensuring accurate measurements across a wide concentration spectrum. Most oxylipins displayed outstanding linearity with R^2^ values exceeding 0.99, with the exception of (±)8-HDHA, which had a slightly lower R^2^ value of 0.9818.

The recovery rates and matrix effects of various oxylipins at three concentrations (low, medium, and high) are shown in Table 3, which lists the precursor PUFAs and a representative oxylipin group. The recovery rates for oxylipins ranged from approximately 70% to 120%, with most values falling between 85% and 110%, indicating a high extraction efficiency. The matrix effect evaluates the impact of plasma components on the analyte ionization efficiency, showing that the majority of analytes had matrix effects between 90% and 110%. However, a few analytes, such as AA and EPA, displayed matrix effects of approximately 70% at medium and high concentrations, indicating ion suppression. Overall, this method ensures reliable quantification of oxylipins in plasma samples.

The accuracy and precision data obtained from the intra-day and inter-day analyses are detailed in Table 4. We utilized three different concentrations to evaluate the four precursors and representative oxylipins. The majority of the analytes exhibited intra-day and inter-day accuracies ranging from 75% to 110%. The precision was expressed as RSD, which was mostly within 15%. These results confirmed that the method is robust, dependable, and reproducible for the quantification of oxylipins in plasma samples.

### 3.3. Analysis of Oxylipin and PUFA Levels in Children’s Plasma

The method was successfully applied to determine the baseline concentrations of the target oxylipins in the plasma of children aged 9–12 years. Quality control samples (pooled plasma and IS) were injected eight times during continuous injection. The results demonstrated that the RSDs of the five IS were below 20%, indicating that the analytical method and instrumentation system exhibited excellent repeatability and stability during sample injection. The extracted ion chromatograms (EICs) of oxylipins and PUFAs in plasma and standards are displayed in Figure 3B, and the similar retention times observed in plasma and standards indicate the consistency of this method. Analytes of four PUFAs and four oxylipins (EPA, DHA, AA, LA, (±)12(13)-DiHOME, (±)9(10)-DiHOME, 9(S)-HODE, 13(S)-HODE) were quantified and analyzed based on age, sex, and body mass index (BMI) status. The concentrations of oxylipins and PUFAs for individual samples are provided in Supplementary Materials Table S1.

### 3.4. Analysis of Oxylipin and PUFA Levels in Children’s Plasma Samples Across Age Groups

**Figure 4:**
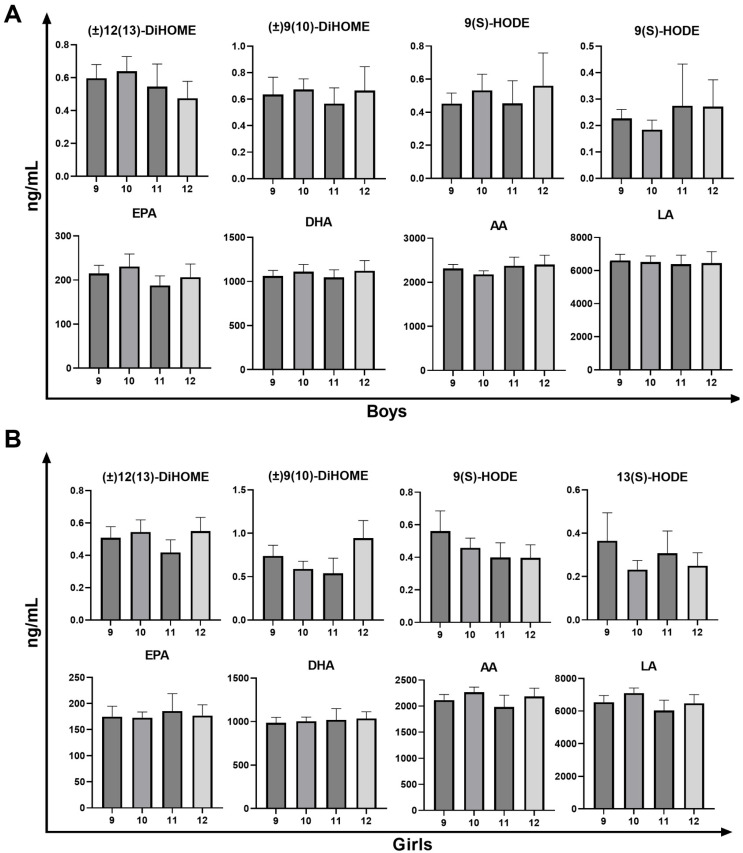
Quantitative amounts of oxylipins and PUFAs in boys (**A**) and girls (**B**) of different age groups (the Kruskal–Wallis test followed by Dunn’s multiple comparisons test was used for comparative analysis of the values, presented as mean ± standard error of the mean). HODE: Hydroxy-octadecadienoic acid; EPA: eicosapentaenoic acid; LA: linoleic acid; AA: arachidonic acid; PUFA: polyunsaturated fatty acid; DiHOME: dihydroxy-octadecenoic acid.

The plasma levels of oxylipins and PUFAs in children aged 9–12 years are compared in Figure 4, and their quantified concentrations are detailed in Table 5. Values are reported as median (interquartile range). Overall, the oxylipin concentrations were exceedingly low, with most values falling below 1 ng/mL. By contrast, the levels of PUFAs were several orders of magnitude higher, ranging from hundreds to thousands of ng/mL. Across all age groups, the LA exhibited the highest concentration of analytes. No significant age-related differences were observed between groups. (±)12(13)-DiHOME and (±)9(10)-DiHOME, which are derived from LA by cytochrome P450, play critical roles in inflammation, immune response, and vascular function [29,30]. While another study indicated that n-6 oxylipins, such as 9,10-DiHOME and 12-HETE, increased with age in healthy children aged 1–17 years [8], the narrower age range of our cohort (9–12 years) likely limited the potential for detecting age-related changes, with a median of approximately 0.5 ng/mL. These differences might be due to age-specific stability within the 9–12 age range or ethnic differences, as our study involved children from Hokkaido, Japan, whereas the other study focused on non-Hispanic Caucasians [8].

Similarly, 9(S)-HODE and 13(S)-HODE were not significantly different between the age groups. Oxylipins derived from LA via the lipoxygenase and cyclooxygenase pathways are markers of oxidative stress and inflammation [31,32]. Although studies correlating HODE levels with human age are lacking, research on middle-aged and aged mice indicated that aging hepatocytes produce 13-HODE, which inhibits catalase activity and leads to liver steatosis [33]. The concentrations of the four precursor PUFAs—EPA, DHA, AA, and LA—were orders of magnitude higher than those of the oxylipins, yet no significant age-related differences were observed. EPA and DHA are essential omega-3 fatty acids for cognitive development and anti-inflammatory processes and are significantly influenced by dietary intake, particularly fish consumption [34,35]. Other studies have shown that the plasma phospholipid concentrations of DHA and EPA are positively correlated with age in older adults [36]. The narrow age range of the participants in this study likely masked significant differences, suggesting consistent EPA and DHA levels within similar age groups. AA and LA are omega-6 fatty acids. Studies have shown that, while AA concentrations remain relatively stable with age, LA tends to decline [37,38]. In this study, LA had the highest concentration among the four precursor PUFAs, which may explain why oxylipins derived from LA were detected at higher concentrations and more easily. Although the observed differences in oxylipin and PUFA levels were not statistically significant across age groups, the established baseline concentrations in this pediatric population provide a valuable reference for future diagnostic studies.

### 3.5. Analysis of Oxylipin and PUFA Levels in Children’s Plasma Samples Across Sexes

**Figure 5:**
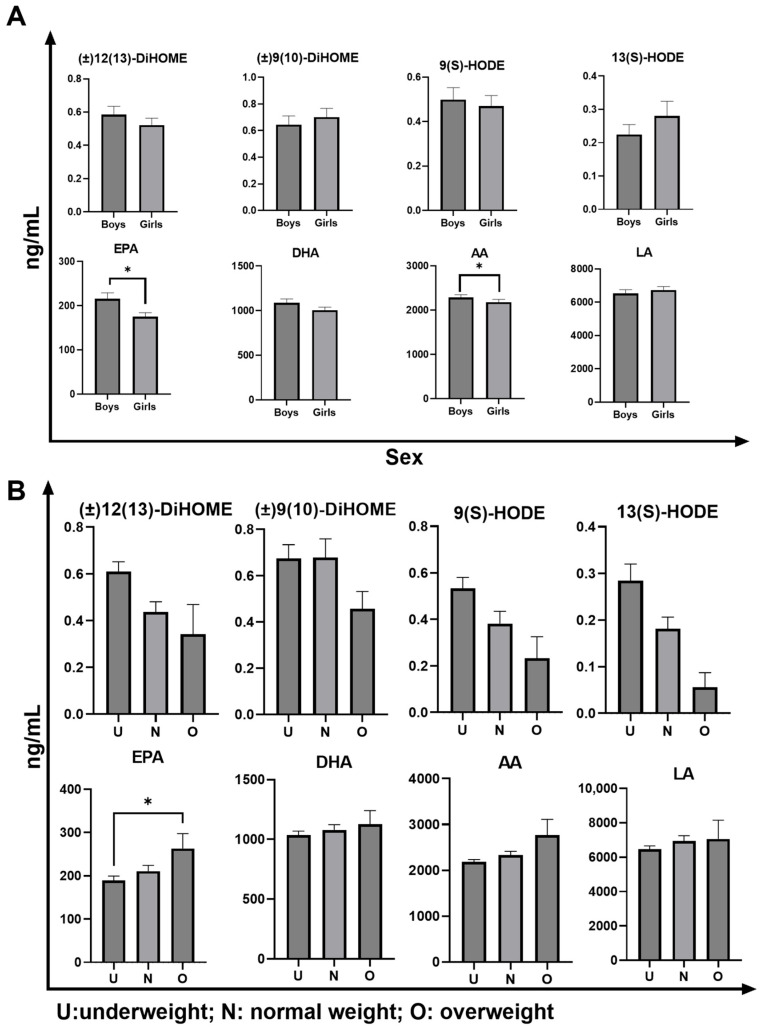
Oxylipin and PUFA levels in plasma of children aged 9–12 years classified by sex and body mass index categories. (**A**) Comparison of oxylipin and PUFA levels between boys and girls. Plasma concentrations of (±)12(13)-DiHOME, (±)9(10)-DiHOME, 9(S)-HODE, 13(S)-HODE, EPA, DHA, AA, and LA were measured. Significant differences were observed in the levels of EPA and AA, with boys showing higher levels compared with those of girls (the Kolmogorov–Smirnov test was conducted for comparative analysis of the values presented as mean ± standard error of the mean, with significance set at * *p* < 0.05). (**B**) Comparison of oxylipin and PUFA levels among the different body mass index categories (U, underweight; N, normal weight; O, overweight). Plasma concentrations of (±)12(13)-DiHOME, (±)9(10)-DiHOME, 9(S)-HODE, 13(S)-HODE, EPA, DHA, AA, and LA were analyzed. Significant differences were observed in the EPA levels, with underweight children showing lower EPA concentrations than those of overweight children (the Kruskal–Wallis test followed by Dunn’s multiple comparisons test was used for comparative analysis of the values, presented as mean ± standard error of the mean, with significance levels denoted as * *p* < 0.05). HODE: Hydroxy-octadecadienoic acid; EPA: eicosapentaenoic acid; LA: linoleic acid; AA: arachidonic acid; PUFA: polyunsaturated fatty acid; DiHOME: dihydroxy-octadecenoic acid.

A comparison of the concentrations of the four oxylipins and four PUFAs between the sexes is presented in Figure 5A. No significant sex differences were observed in oxylipin concentrations. However, boys had significantly higher EPA and AA among the PUFAs. In contrast to our findings, previous studies on fasting plasma have reported higher levels of 12,13-DiHOME in women than in men. This discrepancy may be attributed to age differences between study populations, as previous studies involved older individuals (41.3 ± 5.9 years) [39]. Additionally, studies focusing on proinflammatory exercise patterns found no differences in 9-HODE and 13-HODE levels at rest, which is consistent with our findings; however, female runners showed greater increases post-exercise than male runners [40].

A large population-based study in New Zealand involving approximately 3000 participants over the age of 15 reported lower EPA and higher DHA proportions in women [34], whereas a Scottish study involving 4114 individuals aged 40–59 years found higher DHA levels in the adipose tissue of women than in men [41]. Our study corroborates these findings, as girls had significantly lower EPA levels but no significant difference in DHA levels. Furthermore, changes in AA and LA levels contrast with other studies that have reported higher levels of these lipids in women’s total plasma [42]. Our results showed that girls had lower AA levels than boys, with no significant difference in LA, suggesting that regional, dietary, or ethnic factors may influence these lipid levels.

### 3.6. Analysis of Oxylipin and PUFA Levels in Children’s Plasma Samples Across BMI Categories

The concentrations of the four oxylipins and four PUFAs across various BMI ranges (underweight, normal weight, and overweight) are shown in Figure 5B. The data indicated no significant differences in oxylipin concentrations among the groups, suggesting that these metabolites may not be influenced by body weight in children aged 9–12 years. However, a significant difference was observed in the EPA levels, with overweight children exhibiting higher EPA concentrations than those of underweight children. In a study of 163 adults not taking fish oil supplements, fish consumption and BMI significantly impacted the omega-3 index; each additional fish serving per month increased the index by 0.24 units, while an increase of three BMI units decreased the index by 0.3 units [43]. Given the high fish consumption in the Japanese diet, dietary influences may outweigh the impact of BMI, leading to higher EPA levels in the overweight group. Although oxylipin levels did not vary significantly with BMI, the observed elevation of EPA in overweight children may have diagnostic relevance, particularly in dietary or metabolically driven inflammatory states, and could contribute to the development of diagnostic frameworks.

### 3.7. Analysis of Oxylipin and PUFA Levels in Children’s Plasma Samples in Relation to Blood Test Results

Blood tests were conducted to assess various parameters, including white blood cell count, red blood cell count, hemoglobin, hematocrit, mean corpuscular volume, mean corpuscular hemoglobin, mean corpuscular hemoglobin concentration, platelet count, basophils, eosinophils, neutrophils, lymphocytes, and monocytes. Analysis of the relations between these blood parameters and the oxylipin and PUFA levels revealed significant differences (Supplementary Materials Figure S1). Although comparability between different blood parameters may be limited, a general trend was observed where oxylipins like HODE decreased as blood values such as hemoglobin and monocyte percentage increased, whereas PUFAs showed an increase with platelet count, hematocrit, white blood cell count, red blood cell count, and neutrophil percentage. Given the scarcity of studies on the relationship between lipids and blood parameters, these results support their potential use as diagnostic biomarkers in pediatric health assessments and offer an essential starting point for future research.

### 3.8. Comparison of Oxylipin Extraction Methods

While numerous established LC-MS/MS methods employing acidification and solid-phase extraction (SPE) have demonstrated excellent sensitivity and broad oxylipin coverage, our study proposes a simplified and precise alternative workflow specifically designed for the efficient detection of oxylipins. This method utilizes protein precipitation combined with antioxidant protection (BHT) rather than acidification and SPE, providing a faster and more user-friendly approach that is particularly suitable for high-throughput or exploratory studies.

It is important to note that although many validated SPE protocols are available, these often rely on different types of chromatographic sorbents, and the recovery of oxylipins can vary significantly depending on both the chemical nature of the compound and the specific SPE column employed. Given the structural diversity of oxylipins, including hydroxy, epoxy, and hydroperoxide derivatives—their interactions with SPE media are heterogeneous. As a result, certain oxylipins are efficiently retained and eluted on specific columns, while others may exhibit poor recovery under the same conditions. A previous comparative study involving six different SPE protocols revealed marked discrepancies in the measured plasma concentrations of certain oxylipin families [44]. This variability makes method standardization challenging, particularly when aiming for comprehensive coverage across oxylipin families.

In contrast, the protein precipitation approach offers a broader and more uniform recovery profile, as it does not rely on selective retention mechanisms. When combined with BHT, it enables the detection of both stable and labile oxylipin species.

### 3.9. Constraints and Limitations in This Study

In this study, we employed a methanol protein precipitation extraction method, which offers significant advantages in terms of labor and time efficiency, as well as substantial cost savings compared with that of the SPE method. However, this approach also has some drawbacks, such as lower selectivity than that of SPE, resulting in higher levels of impurities in the samples and reduced analytical sensitivity. We used BHT, a commonly used antioxidant; however, some studies have employed a combination of antioxidants, such as triphenylphosphine and ethylenediaminetetraacetic acid, which could more effectively prevent sample degradation [45]. For biological samples, we used plasma from non-fasting children. It is impractical to require fasting in children, which means that the most recent meal may have influenced the results. Due to the limited age range of our study cohort, this research may not reveal age-related differences in analyte concentrations that might be more evident in a wider age range. Further studies involving a wider age range and diverse populations are necessary to elucidate potential age-related variations.

## 4. Conclusions

In conclusion, we developed a simple and highly selective targeted liquid chromatography–tandem mass spectrometry method that covers 64 oxylipins; provides a foundation for large-scale quantification; exhibits good linearity, accuracy, precision, and reproducibility; and supports diagnostic research and advances insights into physiological health and biomarker discovery. The selection of fragmentation patterns for various isomers was optimized to enhance selectivity. This method was applied to plasma samples from children aged 9–12 years to provide new insights into the distribution of oxylipins and their precursor PUFAs in plasma. The relations between these concentrations and factors, such as age, sex, and BMI, were also explored, and no significant differences were observed in oxylipins with respect to these variables. However, boys had higher levels of EPA and AA than girls, and the dietary increase in EPA appeared to be more significant than the reduction in EPA associated with obesity. These findings not only provide baseline oxylipin profiles in a pediatric population but also highlight the potential utility of this method for early diagnosis and monitoring of inflammation-related conditions in children.

## Figures and Tables

**Table 1:** Mass spectrometry (MS) parameters of oxylipins, polyunsaturated fatty acids (PUFAs), and internal standard.

Analytes	Precursor Ion [M−H]^−^ (*m/z*)	Product Ion (*m/z*)	Collision Energy (eV)	Tube Lens (V)
Prostaglandin F_3α_	351.218	193.1	20	65
Prostaglandin E_3_	349.202	269.2	15	60
8-iso Prostaglandin F_2α_	353.234	193.0	27	81
(±)5-iPF_2α_-VI	353.233	335.1	18	75
Prostaglandin F_2α_	353.232	193.1	22	89
(±)5(6)-DiHETE	335.221	145.0	15	61
(±)19(20)-DiHDPA	361.236	229.4	20	63
(±)13(14)-DiHDPA	361.238	233.1	15	65
9(S)-HOTrE	293.211	171.1	15	64
(±)16(17)-DiHDPA	361.237	193.1	15	67
13(S)-HOTrE	293.212	195.0	15	66
(±)18-HEPE	317.212	215.2	15	65
(±)10(11)-DiHDPA	361.239	153.2	15	63
13(S)-HODE	295.228	195.0	15	66
9(S)-HODE	295.227	171.0	15	65
13-OxoODE	293.214	113.0	15	73
(±)17(18)-EpETE	317.209	215.0	15	66
(±)20-HDHA	343.226	285.0	15	70
9-OxoODE	293.213	185.0	20	68
(±)15-HETE	319.229	219.1	16	67
15-OxoETE	317.213	139.0	17	75
(±)8-HDHA	343.227	125.0	20	62
(±)14(15)-EpETE	317.211	248.0	15	65
(±)16-HDHA	343.230	261.0	15	65
17(S)-HDHA	343.229	245.0	15	63
(±)11-HETE	319.228	167.0	18	74
(±)13-HDHA	343.228	221.0	15	69
(±)11(12)-EpETE	317.210	195.0	15	62
(±)8(9)-EpETE	317.214	155.0	15	69
(±)17(18)-DiHETE	335.221	203.0	15	63
(±)11(12)-DiHETE	335.222	167.0	15	67
(±)8(9)-DiHETE	335.223	185.0	15	64
(±)14(15)-DiHETE	335.224	111.0	20	68
(±)12(13)-DiHOME	313.238	183.0	20	62
(±)9(10)DiHOME	313.239	201.0	20	64
13(S)-HOTrE(γ)	293.212	193.0	15	67
15(S)-HEPE	317.214	219.1	20	67
(±)11-HEPE	317.212	195.1	15	63
(±)7(8)-DiHDPA	361.238	127.1	20	62
(±)8-HEPE	317.210	155.1	15	62
12(S)-HEPE	317.213	207.1	17	75
(±)9-HEPE	317.211	123.1	15	66
5(S)-HEPE	317.209	129.1	20	68
(±)10-HDHA	343.226	181.0	15	66
14(S)-HDHA	343.230	205.0	15	67
(±)8-HETE	319.227	155.0	16	60
(±)12-HETE	319.229	135.0	17	71
12-OxoETE	317.216	153.0	17	72
(±)11-HDHA	343.223	165.0	15	62
(±)7-HDHA	343.224	141.0	15	64
(±)19(20)-EpDPA	343.225	241.6	15	66
(±)5-HETE	319.226	115.0	15	60
(±)12(13)-EpOME	295.228	195.0	15	66
(±)9(10)-EpOME	295.227	171.0	15	65
(±)14(15)-EET	319.230	219.1	14	62
(±)4-HDHA	343.231	101.0	15	61
(±)16(17)-EpDPA	343.232	274.4	15	69
5-OxoETE	317.215	203.2	20	79
(±)5(6)-EET	319.225	191.3	15	74
(±)13(14)-EpDPA	343.227	161.3	15	70
(±)11(12)-EET	319.228	208.0	15	75
(±)10(11)-EpDPA	343.228	153.2	15	61
(±)8(9)-EET	319.231	155.0	16	79
(±)7(8)-EpDPA	343.229	112.8	15	65
EPA	301.217	257.2	16	90
DHA	327.233	283.2	14	92
AA	303.233	259.3	18	91
LA	279.233	261.1	23	91
AA-d8	311.283	267.3	16	62
12(S)-HETE-d8	327.278	184.0	15	64
15(S)-HETE-d8	327.277	226.0	15	67
5(S)-HETE-d8	327.279	116.0	15	65
8-isoProstaglandin F_2α_-d4	357.259	197.0	27	76

**Table 2:** Retention time, limit of detection (LOD), limit of quantification (LOQ), and linearity of oxylipins and poly-unsaturated fatty acids (PUFAs).

Analytes	R.T. (min)	LOD (pg)	LOQ (pg)	R^2^	Linear Range (pg)	Slope
Prostaglandin F_3α_	12.36	5	10	0.9966	5–1000	0.01011
Prostaglandin E_3_	12.48	0.5	1	0.9993	1–1000	0.09813
8-iso Prostaglandin F_2α_	12.52	1	2.5	0.9967	2.5–1000	0.05235
(±)5-iPF2α-VI	12.68	2.5	5	0.9988	5–1000	0.05519
Prostaglandin F2α	12.94	0.5	1	0.995	1–1000	0.04031
(±)17(18)-DiHETE	16.15	5	10	0.995	10–1000	0.0052
(±)11(12)-DiHETE	16.85	0.5	1	0.9979	1–1000	0.03615
(±)8(9)-DiHETE	17.21	1	2.5	0.9995	2.5–1000	0.0175
(±)14(15)-DiHETE	17.22	1	2.5	0.9925	2.5–1000	0.0067
(±)12(13)-DiHOME	17.3	0.5	1	0.9973	1–1000	0.08608
(±)9(10)DiHOME	17.79	0.5	1	0.9987	1–1000	0.13526
(±)5(6)-DiHETE	18.16	2.5	5	0.995	5–1000	0.05946
(±)19(20)-DiHDPA	18.57	2.5	5	0.9921	5–1000	0.00771
(±)13(14)-DiHDPA	19.17	2.5	5	0.9988	5–1000	0.01391
9(S)-HOTrE	19.3	1	2.5	0.9945	2.5–1000	0.02924
(±)16(17)-DiHDPA	19.52	0.5	1	0.9987	1–1000	0.02587
13(S)-HOTrE	19.66	2.5	5	0.9955	2.5–1000	0.01063
(±)18-HEPE	19.81	1	2.5	0.9995	2.5–1000	0.02875
(±)10(11)-DiHDPA	19.89	1	2.5	0.9979	2.5–1000	0.02351
13(S)-HOTrE(γ)	20.14	1	2.5	0.9993	2.5–1000	0.04092
15(S)-HEPE	20.64	2.5	5	0.996	5–1000	0.00493
(±)11-HEPE	20.75	1	2.5	0.9989	2.5–1000	0.02179
(±)7(8)-DiHDPA	20.84	5	10	0.9953	10–1000	0.02686
(±)8-HEPE	20.98	2.5	5	0.9959	5–1000	0.1022
12(S)-HEPE	21.23	25	50	0.9926	50–1000	0.00735
(±)9-HEPE	21.46	5	10	0.9947	10–1000	0.00917
5(S)-HEPE	21.93	1	2.5	0.9958	2.5–1000	0.06759
13(S)-HODE	22.43	0.5	1	0.9977	1–1000	0.05315
9(S)-HODE	22.55	0.25	0.5	0.9991	0.5–1000	0.07289
13-OxoODE	22.67	2.5	5	0.9954	5–1000	0.00611
(±)17(18)-EpETE	22.71	2.5	5	0.9978	5–1000	0.027
(±)20-HDHA	22.97	10	25	0.9967	25–1000	0.00982
9-OxoODE	23.28	2.5	5	0.9974	5–1000	0.06133
(±)15-HETE	23.28	2.5	5	0.9988	5–1000	0.0156
15-OxoETE	23.45	10	25	0.9983	25–1000	0.00851
(±)8-HDHA	23.54	25	50	0.9818	50–1000	0.00401
(±)14(15)-EpETE	23.56	2.5	5	0.994	5–1000	0.01266
(±)16-HDHA	23.56	25	50	0.9965	50–1000	0.00359
17(S)-HDHA	23.68	5	10	0.9988	10–1000	0.00847
(±)11-HETE	23.75	0.5	1	0.9993	1–1000	0.06515
(±)13-HDHA	23.79	1	2.5	0.9904	2.5–1000	0.01295
(±)11(12)-EpETE	23.8	5	10	0.9924	10–1000	0.00641
(±)8(9)-EpETE	23.92	2.5	5	0.9983	5–1000	0.03193
(±)10-HDHA	24.02	2.5	5	0.9995	5–1000	0.01167
14(S)-HDHA	24.03	1	2.5	0.9997	2.5–1000	0.01364
(±)8-HETE	24.1	1	2.5	0.9989	2.5–1000	0.07756
(±)12-HETE	24.11	5	10	0.996	10–1000	0.00474
12-OxoETE	24.18	2.5	5	0.9981	5–1000	0.01544
(±)11-HDHA	24.26	2.5	5	0.9929	2.5–1000	0.00515
(±)7-HDHA	24.37	2.5	5	0.9981	5–1000	0.03476
(±)5-HETE	24.57	0.625	1.5625	0.9972	1.5625–1000	0.05294
(±)12(13)-EpOME	24.76	0.1	0.25	0.9974	0.25–1000	0.15922
(±)19(20)-EpDPA	24.83	5	10	0.9923	10–1000	0.00335
(±)9(10)-EpOME	24.88	1	2.5	0.9997	2.5–1000	0.09169
(±)14(15)-EET	24.94	2.5	5	0.9986	2.5–1000	0.01247
(±)4-HDHA	25.08	2.5	5	0.9986	5–1000	0.06812
(±)16(17)-EpDPA	25.2	2.5	5	0.9987	5–1000	0.00355
5-OxoETE	25.28	5	10	0.9959	10–1000	0.05103
(±)13(14)-EpDPA	25.33	5	10	0.9936	10–1000	0.00624
(±)11(12)-EET	25.42	10	25	0.9949	25–1000	0.00654
(±)10(11)-EpDPA	25.45	2.5	5	0.9966	5–1000	0.02115
(±)8(9)-EET	25.54	10	25	0.9962	25–1000	0.02073
(±)7(8)-EpDPA	25.58	10	25	0.9979	25–1000	0.00974
(±)5(6)-EET	25.66	5	10	0.9943	10–1000	0.01971
EPA	26.78	10	20	0.9992	20–200,000	0.00549
DHA	27.51	5	10	0.9998	10–200,000	0.01596
AA	27.71	10	20	0.9997	20–200,000	0.00134
LA	27.81	10	20	0.9976	20–200,000	0.00043

**Table 3:** Recovery and matrix effects of oxylipins and polyunsaturated fatty acids (PUFAs).

	50 ng/mL		75 ng/mL		100 ng/mL	
Analytes	Recovery	Matrix Effect (%)	Recovery	Matrix Effect (%)	Recovery	Matrix Effect (%)
	(%)		(%)		(%)	
AA *	92.5	95.5	96	98.5	106.1	94.9
(±)15-HETE	121.9	97.3	98.8	103.2	94.6	93.3
5-OxoETE	99	110.7	100.6	101.9	100.3	116.8
PGF_2α_	99.4	100.9	101	104.5	105.4	105.7
(±)11(12)-EET	92.4	95.9	108	88.8	121.3	115.8
LA *	92.2	89.7	114.1	108.3	114.5	100.3
13(S)-HODE	102.9	103.8	97.2	110.3	102	96.7
13-OxoODE	84.2	110.5	95.8	109.1	99.5	102.9
(±)9(10)-DiHOME	84.4	103	93.7	104.4	92.8	109.3
(±)9(10)-EpOME	93.8	87.4	83.7	87.5	85.8	85.8
DHA *	89.5	93.6	103.6	106.1	116.3	105.8
(±)4-HDHA	104.5	98.1	122.1	94.5	106.4	107.3
(±)13(14)-EpDPA	87.2	96.1	82.6	91.4	82.5	84.3
(±)7(8)-DiHDPA	90.3	101.6	98.3	98.7	97.1	113.4
EPA *	92.7	94.2	98.6	99.3	108.7	95.4
(±)11-HEPE	95	102.8	96.3	104.5	94.6	107.7
(±)17(18)-EpETE	92.7	99.7	107	111.7	102.8	112.2
(±)17(18)-DiHETE	98.2	102.5	99.2	106.2	99.9	108
13(S)-HOTrE	89.5	99.4	100.8	113.8	98.9	106.9
Mean ± SD (total)	90 ± 13	97 ± 11	95 ± 12	98 ± 14	95 ± 13	90 ± 18

* The concentrations of PUFAs are as follows: low at 5 μg/mL, medium at 10 μg/mL, and high at 20 μg/mL.

**Table 4:** Accuracy and precision of oxylipins and polyunsaturated fatty acids (PUFAs).

	50 ng/mL				75 ng/mL				100 ng/mL			
	Intra-Day (%)		Inter-Day (%)		Intra-Day (%)		Inter-Day (%)		Intra-Day (%)		Inter-Day (%)	
Analytes	Accuracy	Precision	Accuracy	Precision	Accuracy	Precision	Accuracy	Precision	Accuracy	Precision	Accuracy	Precision
AA *	73.9	3.7	85.0	1.8	75.7	4.0	82.9	3.5	90.6	4.2	77.6	2.0
15HETE	103.7	9.4	75.2	8.5	91.0	7.3	81.4	8.2	84.8	4.5	78.5	4.4
5-OxoETE	93.6	8.1	93.5	5.6	90.3	7.6	89.3	6.8	89.9	2.6	85.8	6.0
PGF2α	86.4	3.5	87.0	2.1	90.7	1.5	86.0	1.2	98.3	1.9	92.5	0.8
(±)11(12)-EET	98.4	6.5	99.7	9.4	81.9	14.4	87.7	12.5	88.2	10.8	76.7	6.9
LA *	75.7	6.4	83.4	5.0	79.6	4.2	82.3	6.4	93.6	3.3	76.9	2.1
13(S)-HODE	94.2	5.3	78.0	2.1	89.0	1.4	80.4	4.5	89.9	1.9	85.3	5.5
13-OxoODE	68.5	5.2	79.0	2.8	81.5	5.3	81.1	3.6	83.3	5.4	82.5	7.3
(±)9(10)DiHOME	72.5	4.4	76.4	4.2	76.8	2.8	75.4	2.9	75.0	0.6	75.1	4.9
(±)9(10)-EpOME	89.0	10.2	91.7	8.0	81.9	14.0	80.6	4.9	79.6	11.6	79.5	5.5
DHA *	76.9	3.8	88.9	3.0	87.4	2.0	99.5	2.6	110.7	5.3	92.9	3.9
(±)4-HDHA	104.5	12.4	87.0	0.9	111.0	8.1	86.4	4.7	92.0	11.4	76.5	6.6
(±)13(14)-EpDPA	79.0	12.9	78.6	12.8	77.8	7.1	76.7	6.2	83.4	7.6	74.5	6.6
(±)7(8)-DiHDPA	80.9	4.6	93.5	11.4	89.9	9.7	85.6	5.5	86.6	2.5	88.1	4.4
EPA *	79.4	4.1	88.9	1.5	82.2	3.2	90.0	3.7	104.5	3.0	85.7	2.1
(±)11-HEPE	80.6	10.3	83.8	4.1	80.1	1.6	77.5	4.1	77.5	2.7	78.6	4.2
(±)17(18)-EpETE	97.3	14.8	104.6	8.3	105.4	4.1	101.9	7.5	102.4	6.6	100.8	4.4
(±)17(18)-DiHETE	91.7	2.1	91.4	5.2	88.2	9.1	86.0	3.8	90.1	1.4	92.2	4.9
13(S)-HOTrE	81.2	10.8	82.2	2.5	88.9	5.4	89.0	2.4	86.3	4.3	89.5	3.6

* The concentrations of PUFAs are as follows: low at 5 μg/mL, medium at 10 μg/mL, and high at 20 μg/mL.

**Table 5:** Concentrations of plasma oxylipins and polyunsaturated fatty acids (PUFAs) in boys and girls categorized by age groups. Values are presented as median (interquartile range).

	Boys (ng/mL)				Girls (ng/mL)			
Analytes	9 y	10 y	11 y	12 y	9 y	10 y	11 y	12 y
(Detection Frequency)								
(±)12(13)-DiHOME	0.4	0.5	0.3	0.4	0.4	0.4	0.5	0.5
(83.30%)	(0.2–0.7)	(0.2–0.8)	(0.2–0.7)	(0.1–0.7)	(0.2–0.8)	(0.2–0.6)	(0.2–0.5)	(0.2–0.9)
(±)9(10)-DiHOME	0.3	0.6	0.4	0.4	0.4	0.3	0.2	0.6
(89.20%)	(0.1–0.8)	(0.2–0.9)	(0.3–0.6)	(0.2–0.9)	(0.2–0.9)	(0.2–0.8)	(0.1–0.7)	(0.3–1.0)
13(S)-HODE	0.2	0.1	0.1	0.1	0.2	0.1	0.1	0.2
(58.20%)	(0.0–0.3)	(0.0–0.3)	(0.0–0.2)	(0.0–0.3)	(0.0–0.4)	(0.0–0.2)	(0.0–0.6)	(0.0–0.3)
9(S)-HODE	0.2	0.3	0.1	0.3	0.4	0.2	0.3	0.2
(85.40%)	(0.1–0.6)	(0.1–0.5)	(0.1–0.6)	(0.1–0.5)	(0.2–0.6)	(0.1–0.7)	(0.1–0.6)	(0.1–0.7)
EPA	165	178	165	162	145	142	163	158
(100%)	(100–279)	(108–241)	(108–264)	(124–228)	(101–196)	(106–222)	(108–181)	(90–200)
DPA	908	926	968	1111	905	915	868	1004
(100%)	(738–1320)	(744–1333)	(765–1325)	(727–1304)	(715–1191)	(697–1295)	(729–1105)	(737–1387)
AA	2315	2185	2176	2525	2050	2064	1913	2117
(100%)	(1745–2756)	(1680–2628)	(1658–2708)	(1398–3034)	(1663–2407)	(1756–2562)	(1453–2111)	(1887–2406)
LA	5779	5465	5675	5905	5993	6544	5761	5866
(100%)	(4415–7944)	(4841–7751)	(4862–7932)	(3780–8238)	(4398–8514)	(5210–8584)	(4444–6546)	(4716–8438)

DiHOME: Dihydroxy-octadecenoic acid; HODE: hydroxy-octadecadienoic acid; EPA: eicosapentaenoic acid; LA: linoleic acid; AA: arachidonic acid.

## Data Availability

The data is available from the corresponding author upon reasonable request.

## Supplementary Materials

The following Supplementary Materials can be downloaded at: https://www.mdpi.com/article/10.3390/diagnostics15151870/s1, Table S1: Concentrations of oxylipins and PUFAs for individual samples (ng/mL); Figure S1: Correlation of plasma oxylipins and PUFAs with blood test parameters in children; Figure S2: Extracted ion chromatogram of oxylipins and PUFAs.

## Abbreviations

The following abbreviations are used in this manuscript:

LC-MS	Liquid chromatography–mass spectrometry
EIC	Extracted ion chromatogram
PUFA	Polyunsaturated fatty acid
LOD	Limit of detection
LOQ	Limit of quantification
DHA	Docosahexaenoic acid
EPA	Eicosapentaenoic acid
LA	Linoleic acid
AA	Arachidonic acid
EET	Epoxyeicosatrienoic acid
GC-MS	Gas chromatography–mass spectrometry
DiHOME	Dihydroxy-octadecenoic acid
HODE	Hydroxy-octadecadienoic acid
DiHDPA	Dihydroxy-docosapentaenoic acid
BHT	Butylated hydroxytoluene
RSD	Relative standard deviation
BMI	Body mass index
EpDPA	Epoxy-docosapentaenoic acid
HEPE	Hydroxy-eicosapentaenoic acid
EpETE	Epoxy-eicosatetraenoic acid
DiHETE	Dihydroxy-eicosatetraenoic acid
HDHA	Hydroxy-docosahexaenoic acid
HOTrE	Hydroxy-octadecatrienoic acid
HETE	Hydroxy-eicosatetraenoic acid
OxoETE	Oxo-eicosatetraenoic acid
MRM	Multiple reaction monitoring
SPE	Solid-phase extraction
